# Supplementary material for: Paternal DNA methylation is remodeled to maternal levels in rice zygote
Source: Nat Commun. 2023 Oct 18;14:6571. doi: 10.1038/s41467-023-42394-0 (PMC10584822; doi:10.1038/s41467-023-42394-0)
Supplement: Supplementary file 1 — Supplementary Information [file 41467_2023_42394_MOESM1_ESM.pdf]

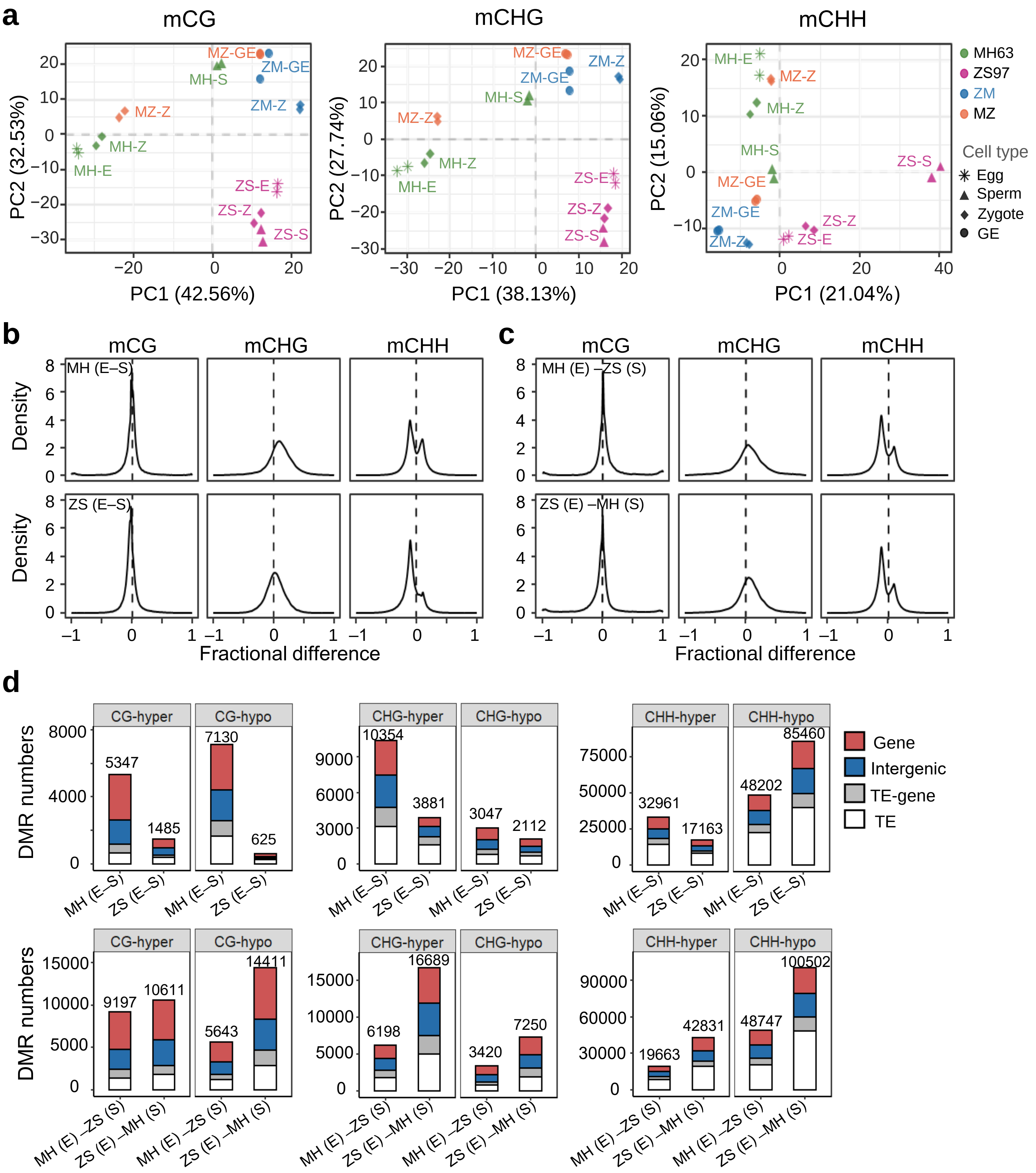

Supplementary Fig. 1. DNA methylomes of inbred and/or hybrid rice gametes, zygotes, and globular embryos. (a) PCA analysis of DNA methylomes of gametes, zygotes and globular embryos (GE) of MH63 and ZS97 inbred and hybrid lines. Different rice varieties are denoted by colors, different cell-types by shapes. **(b)** Density plot of methylation difference at 50-bp windows between sperm and egg of MH63 [MH (E – S),upper panel] and ZS97 [ZS (E – S), lower panel]; **(c)** methylation levels between MH63 egg and ZS97 sperm [MH (E) – ZS (S), upper panel], and between ZS97 egg and MH63 sperm [ZS (E) – MH (S), lower panel]. **(d)** DMR numbers of 50-bp windows of egg versus sperm in MH63 and ZS97(upper, in the same genotypes; lower, between the 2 genotypes). DMRs in gene body, intergenic, TE-genes and TEs are indicated by red, blue, grey and white, respectively.

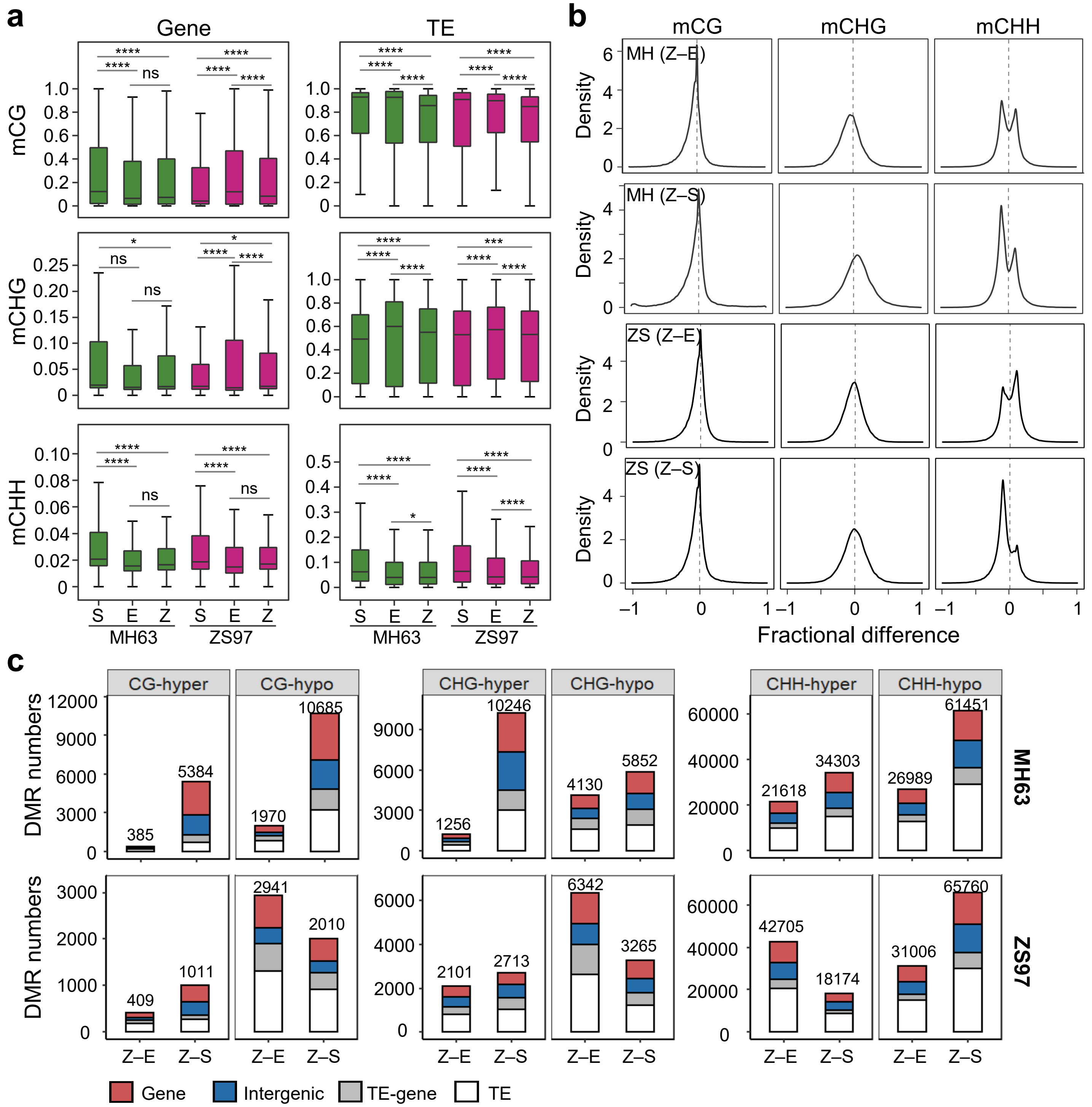

Supplementary Fig. 2. DNA methylation in the gametes and zygotes of the hybrid parental lines. (a) Boxplots showing the CG, CHG, and CHH methylation levels in genes (n = 39,407) and transposable elements (TEs) (n = 375,397) of sperm (S), egg (E) and zygote (Z) cells of the MH63 and ZS97 varieties. The horizontal line within the box represents the median, box limits represent the interquartile range (IQR), and whiskers represent 1.5 × IQR . Values are averages from the two replicates (* P < 0.05, *** P < 0.001, ****P < 0.0001, ns, not significant, two-sided Wilcoxon rank-sum test). n = 2 biologically independent samples for each cell type examined. **(b)** Density plot showing the frequency distribution of methylation difference at 50-bp windows between zygote and egg (Z – E) and between zygote and sperm (Z – S) in MH63 (MH) and ZS97 (ZS). **(c)** Differentially methylated regions (DMRs) between zygote and egg (Z – E), and between zygote and sperm (Z – S) in MH63 (upper panel) and ZS97 (lower panel). DMRs numbers and distributions (gene body, red; intergenic, blue; TE-genes, grey; and TE, white) are indicated. Source data are provided as a Source Data file.

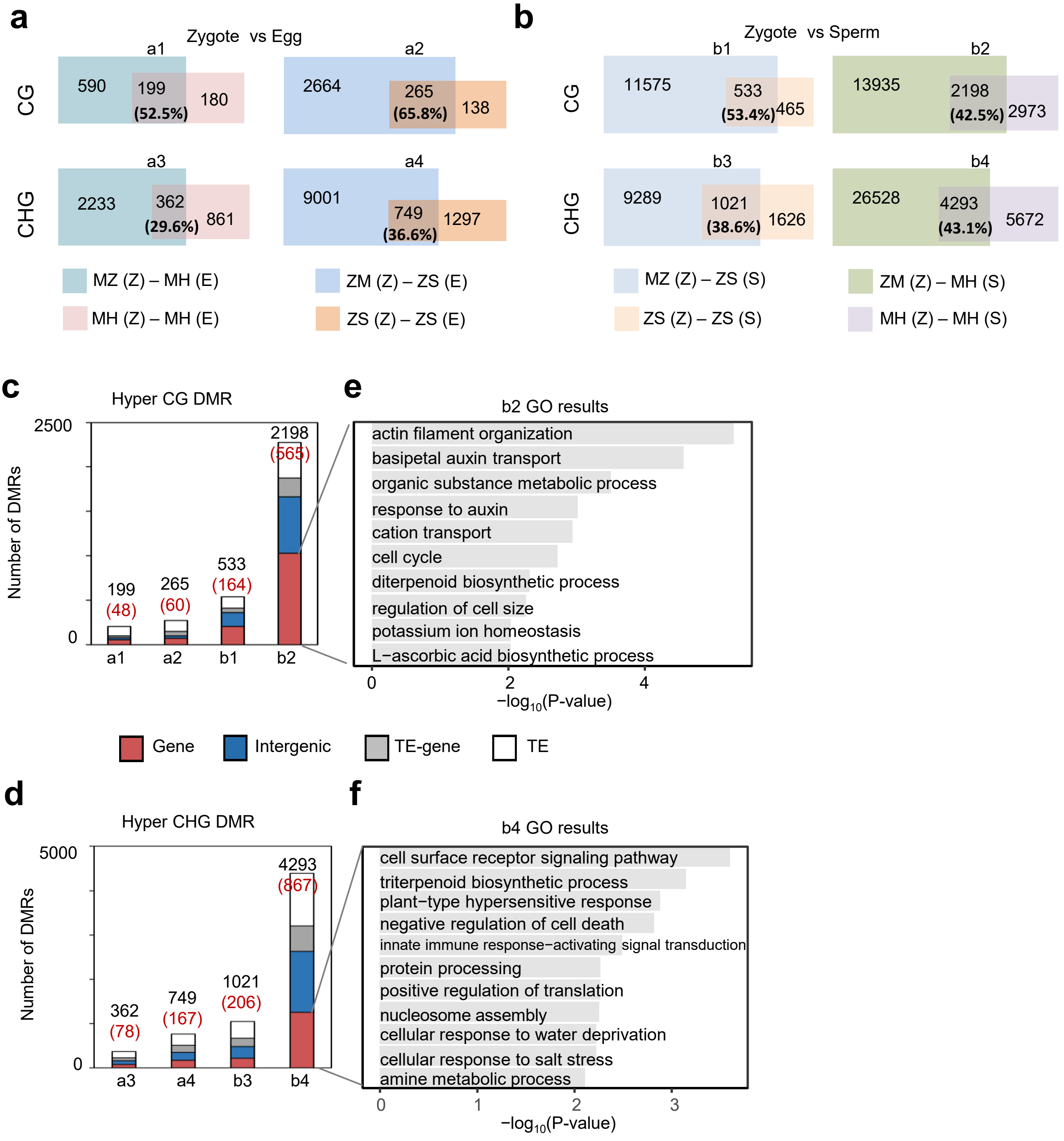

Supplementary Fig. 3. Conserved hyper CG and CHG DMRs between inbred and hybrid zygotes relative to the gametes. **(a)** Venn diagrams showing the overlapping of hyper DMRs between hybrid zygotes (MZ zygote [MZ (Z)], ZM zygote [ZM (Z)]) and inbred zygotes (ZS97 zygote [ZS (Z)], MH63 zygote [MH (Z)]) versus female gametes (ZS97 egg [ZS (E)], MH63 egg [MH (E)]); **(b)** overlapping of hyper DMRs between hybrid zygotes (MZ zygote [MZ (Z)], ZM zygote [ZM (Z)]) and inbred zygotes (ZS97 zygote [ZS (Z)], MH63 zygote [MH (Z)]) versus male gametes (ZS97 sperm [ZS (S)], MH63 sperm [MH (S)]). Genomic distribution of the overlapping CG **(c)** or CHG **(d)** hyper DMRs in **a**, and **b**. a1, a2, b1 and b2 are CG DMRs, a3, a4,b3 and b4 are CHG DMRs. DMRs in gene body, intergenic, TE-gene and TE regions are denoted by red, blue, grey and white, respectively. The numbers of DMR-related genes are in red. **(e) and (f)** GO enrichment of the overlapping hyper CG DMR **(e)** **or** hyper CHG DMR **(f)** related genes in ZM zygote and MH zygote versus MH sperm.

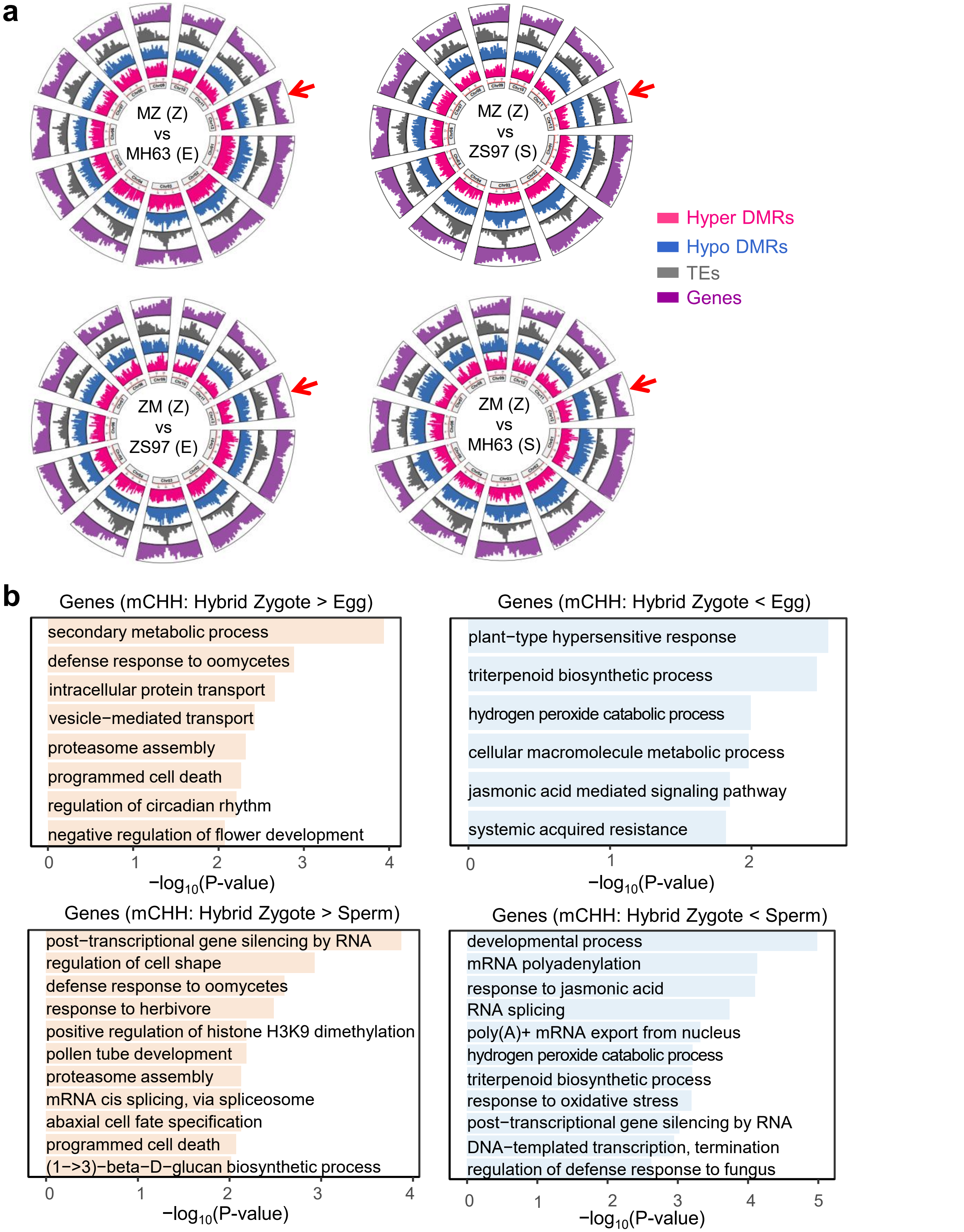

Supplementary Fig. 4. Genomic distribution of the CHH DMRs between hybrid zygotes and male or female gamete. **(a)** Circos plots showing the genomic distribution of the CHH DMRs between hybrid zygotes (MZ and ZM) and male/female gametes (sperm and egg of MH63 or ZS97). Each sector represents each chromosome of rice. Hyper- and hypo-DMRs are denoted by magenta and blue, TE and genic regions are denoted by grey and purple, respectively. Data in circles are displayed in nonoverlapping 10-kb intervals. **(b)** GO enrichment of the differentially methylated genes in CHH context between hybrid zygotes and male/female gametes. Source data are provided as a Source Data file.

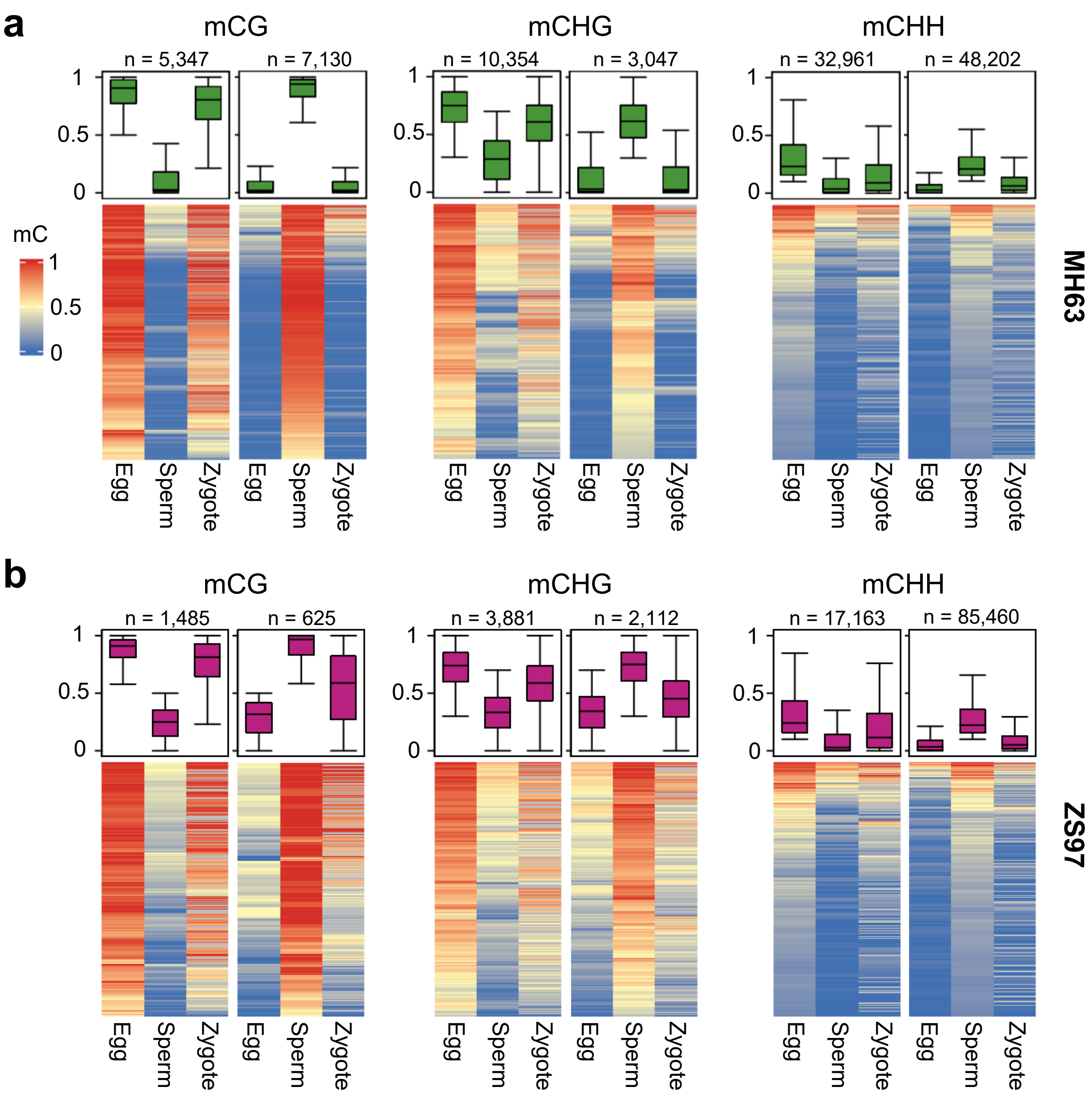

Supplementary Fig. 5. Zygotic methylation levels of the egg versus sperm DMRs in MH63 and ZS97. Boxplots and heatmaps showing the methylation levels of the egg-sperm DMRs in egg, sperm, and zygote of MH63 **(a)** and ZS97 **(b)**. The numbers (N) of DMRs between egg and sperm at CG, CHG, and CHH contexts are indicated.). n = 2 biologically independent samples for each cell type examined. The horizontal line within the box represents the median, box limits represent the interquartile range (IQR), and whiskers represent 1.5 × IQR . Source data are provided as a Source Data file.

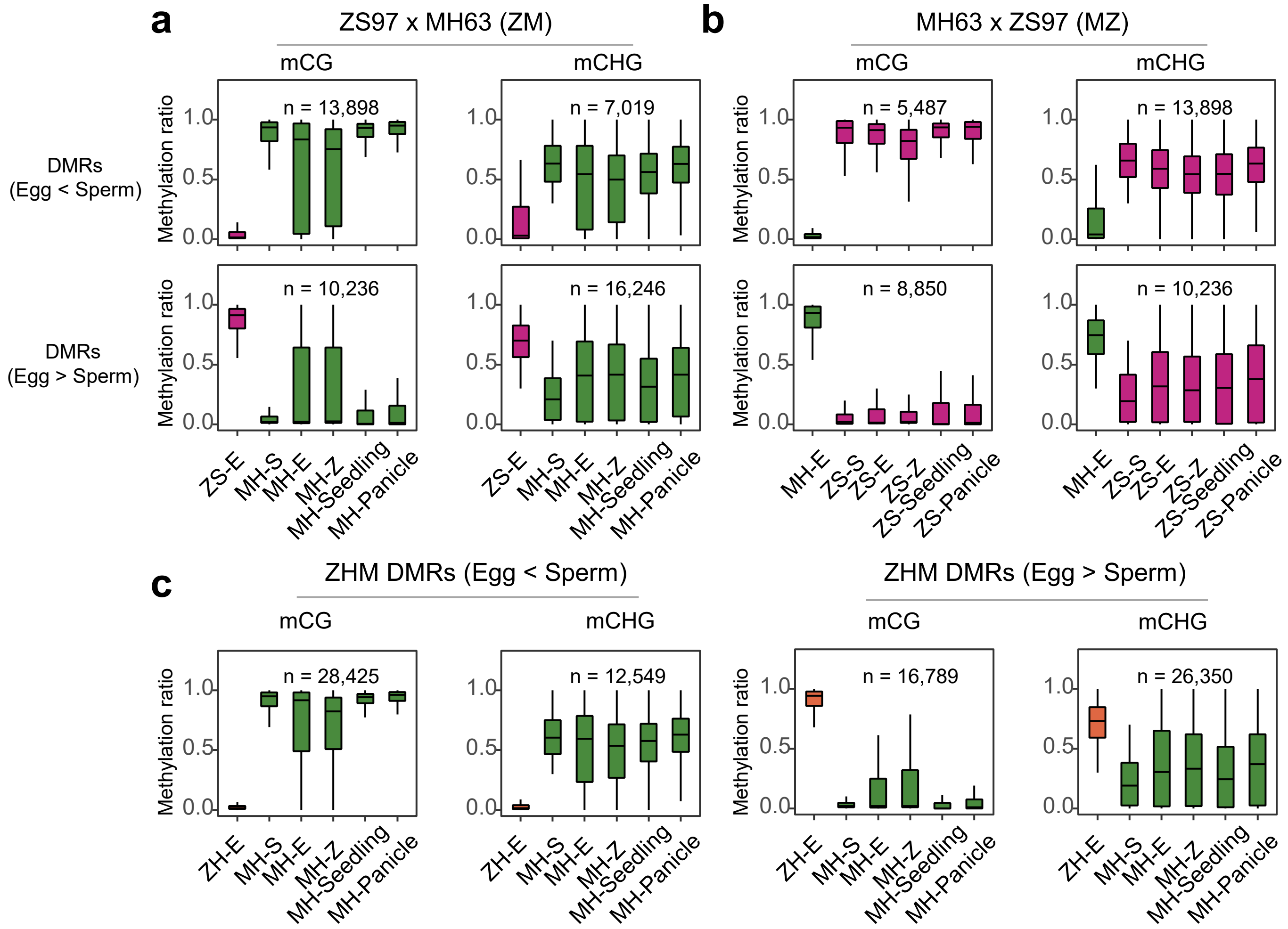

Supplementary Fig. 6. DNA methylation levels of the DMRs between hybrid parental gametes in sperm, egg, zygote and vegetative tissues of the paternal lines. **(a)** Boxplots showing the DNA methylation levels of the DMRs between MH63 sperm and ZS97 egg in sperm, egg, zygote, seedling, and panicle of MH63. **(b)** Boxplots showing the methylation levels of the DMRs between ZS97 sperm and MH63 egg in sperm, egg, zygote, seedling, and panicle of ZS97. **(c)** Boxplots showing the methylation levels of the DMRs between MH63 sperm and ZH11 egg in sperm, egg, zygote, seedling, and panicle of MH63. N denotes the numbers of E – S DMRs.). n = 2 biologically independent samples for each cell type examined. The horizontal line within the box represents the median, box limits represent the interquartile range (IQR), and whiskers represent 1.5 × IQR . Source data are provided as a Source Data file.

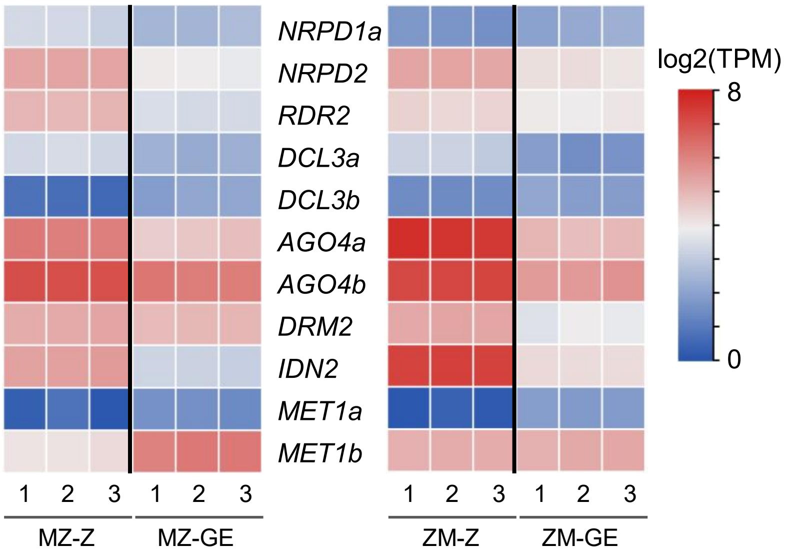

Supplementary Fig. 7. Heatmaps of transcript levels of RdDM pathway genes in rice zygote and globular embryo. Source data are provided as a Source Data file.

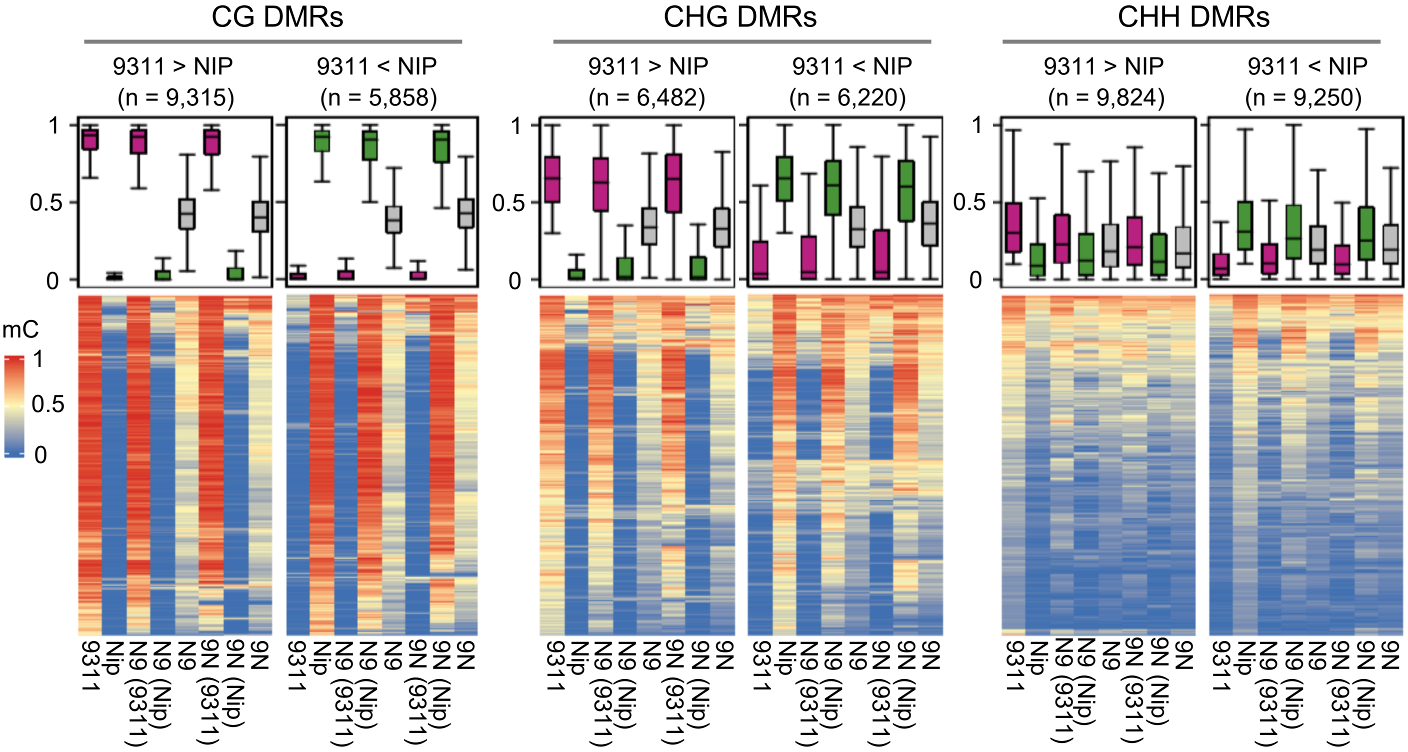

Supplementary Fig. 8. Maintenance of parental allelic-specific methylations in the reciprocal hybrids of NIP and 9311. Boxplots and heatmaps showing the paternal and maternal methylation levels of the DMRs between Nip and 9311 in the reciprocal hybrids N9 (Nip×9311, Nip as female and 9311 as male) and 9N (9311×Nip, 9311 as female and Nip as male). N9 (9311) indicated paternal-allele-specific methylation in NIP×9311, N9 (Nip) indicated maternal-allele-specific methylation in Nip×9311, 9N (Nip) indicate paternal-allele-specific methylation in 9311×Nip, 9N (9311) indicate maternal-allele-specific methylation in 9311×Nip. The numbers (N) of the hyper (9311 > Nip) or hypo (9311 < Nip) DMRs between 9311 and Nip at CG, CHG and CHH contexts are indicated. The horizontal line within the box represents the median, box limits represent the interquartile range (IQR), and whiskers represent 1.5 × IQR. Source data are provided as a Source Data file.

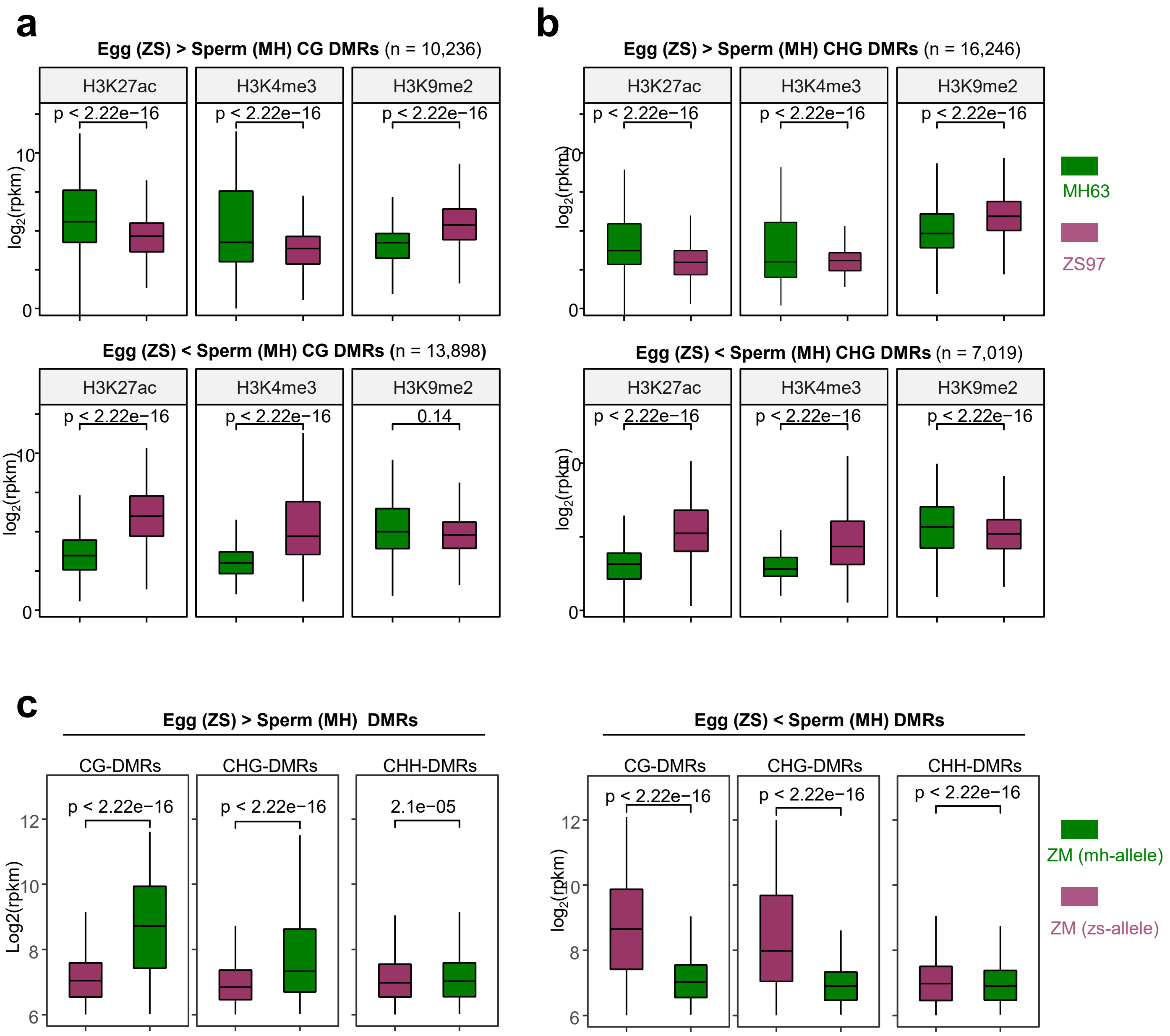

Supplementary Fig. 9. Boxplots of histone modification levels of the egg-sperm DMRs in the parental lines. (a, b) H3K27ac, H3K4me3 and H3K9me2 levels of the CG **(a)** and CHG **(b)** DMRs between ZS97 egg and MH63 sperm in MH63 and ZS97 seedlings. Upper panel, hyper-DMRs (ZS97 egg > MH63 sperm); lower panel, hypo-DMRs (ZS97 egg < MH63 sperm). **(c)** Allelic-specific H3K4me3 level of the CG, CHG, and CHH DMRs between ZS97 egg and MH63 sperm in ZM seedling. left panel, hyper-DMRs (ZS97 egg > MH63 sperm); right panel, hypo-DMRs (ZS97 egg < MH63 sperm). N denotes the numbers of E – S DMRs. The horizontal line within the box represents the median, box limits represent the interquartile range (IQR), and whiskers represent 1.5 × IQR . P-value are calculated by two-sided Wilcoxon rank-sum test. Source data are provided as a Source Data file.

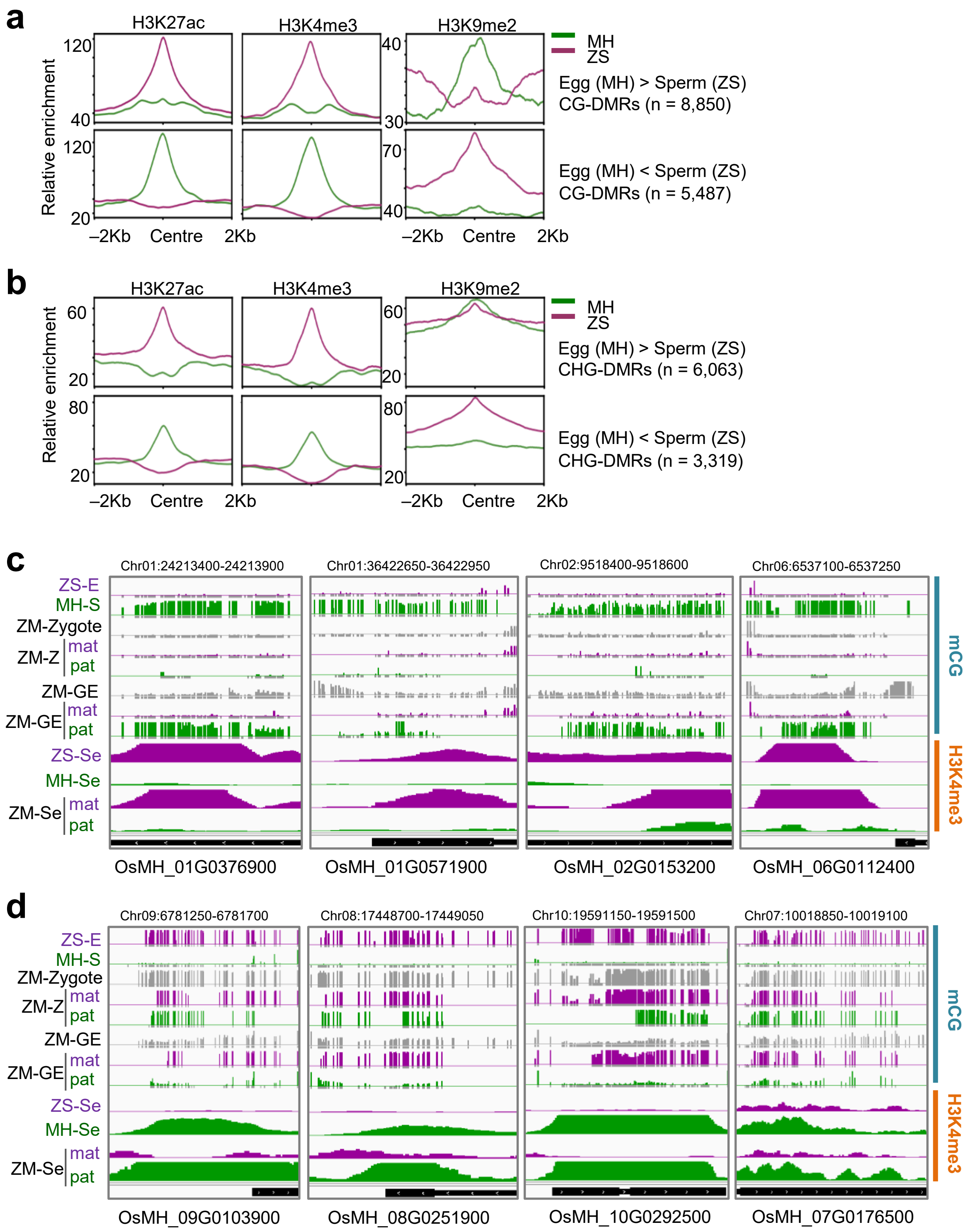

Supplementary Fig. 10. Parental allelic-specific methylations associated with specific histone marks in the parental lines. **(a, b)** H3K27ac, H3K4me3 and H3K9me2 modifications of the CG **(a)** and CHG **(b)** DMRs between MH63 egg and ZS97 sperm MH63 and ZS97 seedlings, upper panel indicate the hyper DMRs (MH63 Egg > ZS97 Sperm), lower panel indicate the hypo DMRs (MH63 Egg < ZS97 Sperm). **(c, d)** Genome browser screenshots showing the paternal (pat) and maternal (mat) allelic-specific methylation in hybrid ZM zygote and globular embryo (GE) and H3K4me3 in MH63, ZS97, and ZM seedling (Se) of the CG hyper **(c)** and hypo **(d)** DMRs between MH63 sperm and ZS97 egg. ZS egg (ZS-E), MH sperm (MH-S), ZM Zygote (ZM-Z), ZM globular embryo (ZM-GE), maternal (mat) and paternal (pat) alleles of ZM Zygote, GE, and seedling (Se). H3K4me3 ChIP-seq signal (RPKM scale 0 to 600). Magenta for data from ZS97, Green for data from MH63.

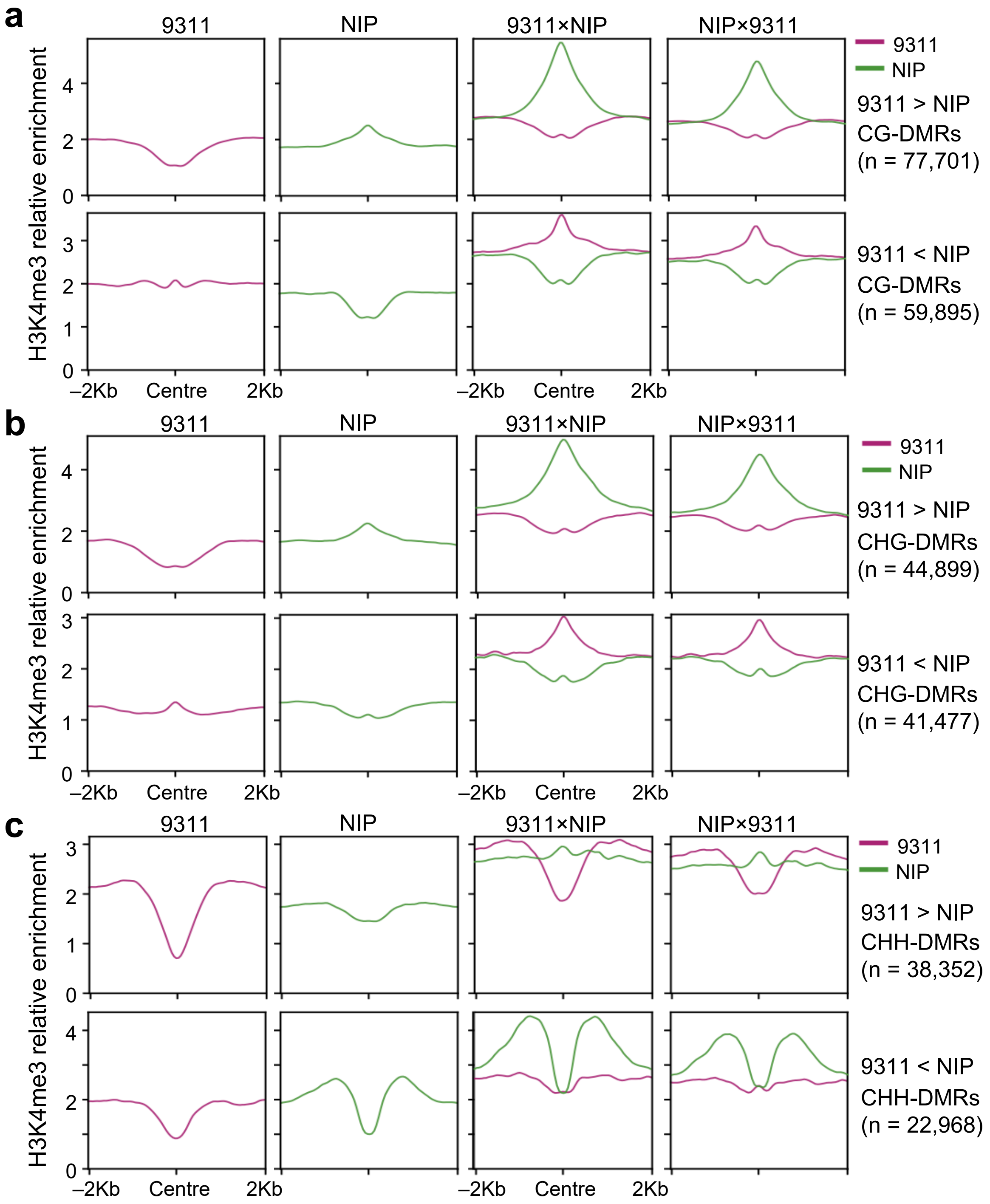

Supplementary Fig. 11. H3K4me3 levels of the DMRs between NIP and 9311 in the parental lines and the reciprocal hybrids. H3K4me3 levels of the CG **(a)**, CHG **(b)**, and CHH **(c)** DMRs between 9311 and NIP in 9311, NIP, 9311×NIP and NIP×9311 plants. Upper panels, hyper-DMRs (9311 > NIP); lower panels, hypo-DMRs (9311 < NIP). In the hybrids, the parental allele-specific H3K4me3 levels are shown.

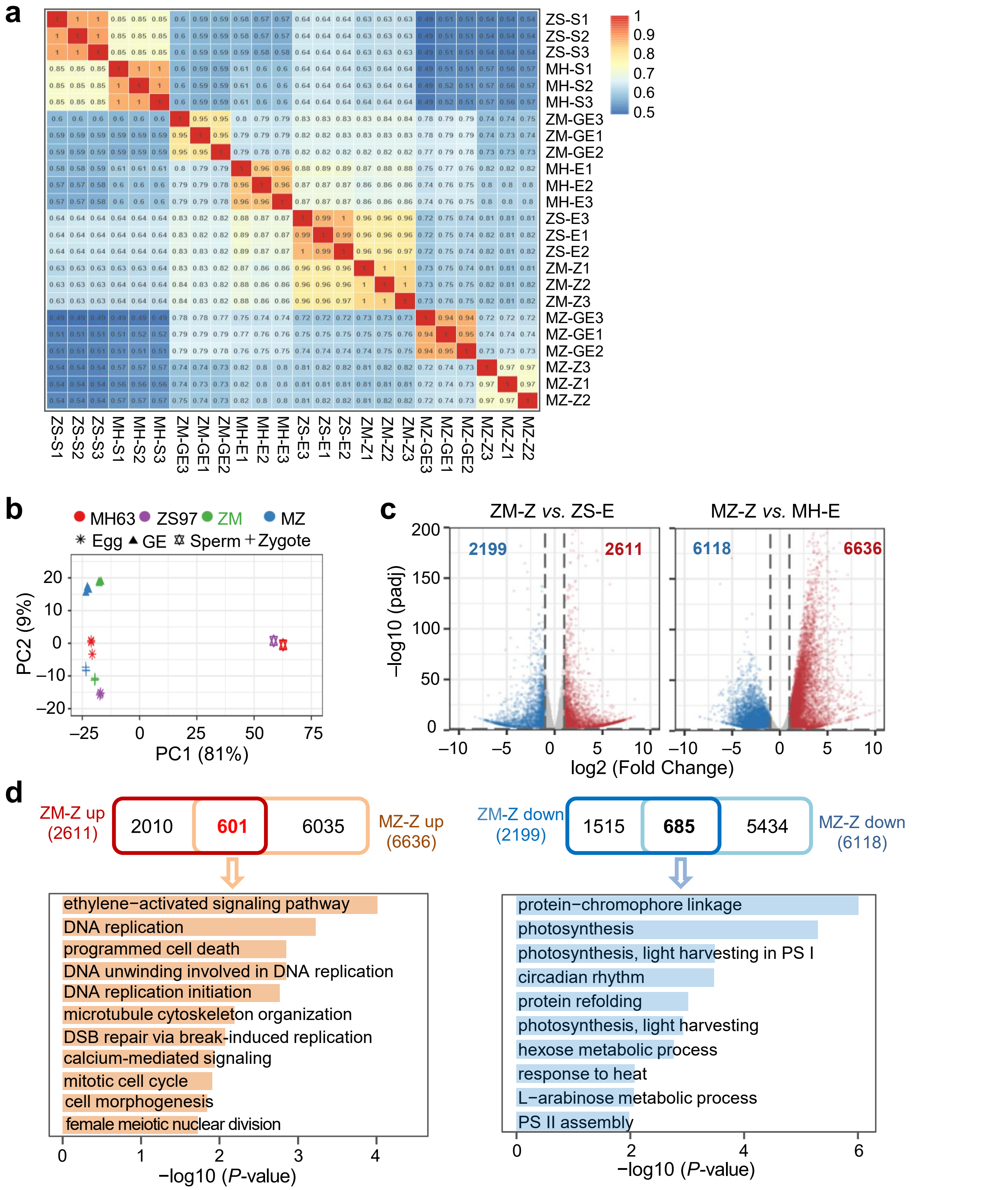

Supplementary Fig. 12. Transcriptomic analysis of the reciprocal hybrid zygotes. (a) Heatmap and Spearman coefficients of RNA-seq replicates of MH63 sperm (MH-S) and egg (MH-E), ZS97 sperm (ZS-S) and egg (ZS-E), ZM zygote (ZM-Z) and globular embryo (ZM-GE), and MZ zygote (MZ-Z) and globular embryo (MZ-GE). Three biological replicates are shown. **(b)** PCA analysis of transcriptomes of MH63 and ZS97 sperm and egg cell and the reciprocal hybrids (ZM and MZ) zygotes as well as globular embryos. For each sample, three biological replicates were produced. **(c)** Volcano-plot showing the differentially expressed genes (DEGs) identified in ZM zygote versus ZS97 egg (upper), MZ zygote versus MH63 egg (lower). |log2 (Fold Change)| > 1 and p-adj < 0.01.). n = 3 biologically independent samples for each cell type examined. **(d)** Venn diagrams and GO enrichment analysis of the commonly up-regulated (n = 601) and downregulated (n = 684) genes in hybrid zygotes versus eggs (ZM zygote versus ZS97 egg and MZ zygote versus MH63 egg).

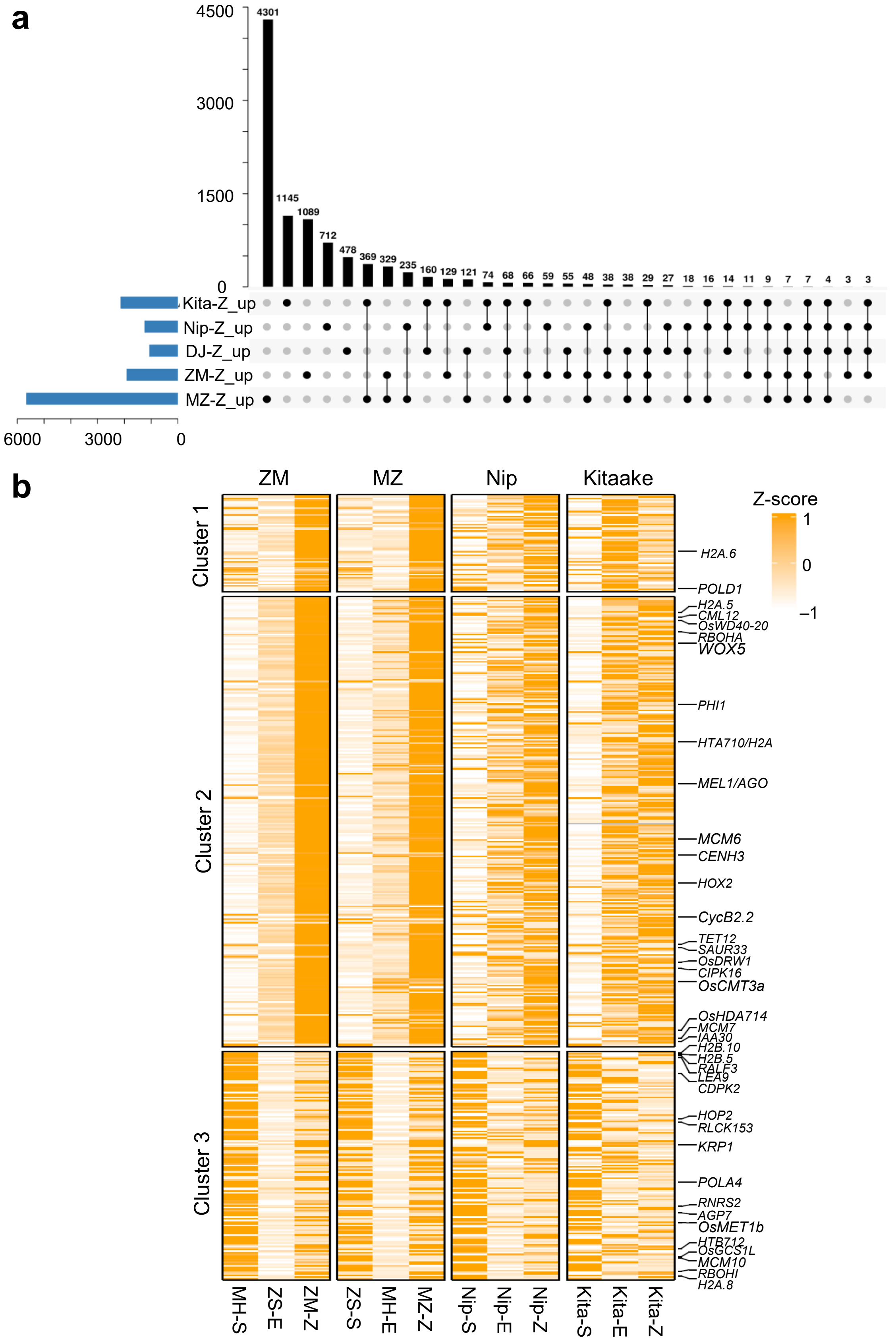

Supplementary Fig. 13. Analysis of hybrid zygote transcriptomes. **(a)** Upset-plot showing the overlapping up-regulated genes in Kitaake (Kita-Z, Anderson et al., 2017), Nipponbare (Nip-Z, Rahman et al., 2019), Donjin (DJ-Z, Zhou et al., 2021), ZM (ZM-Z) and MZ (MZ-Z) zygotes versus egg. **(b)** Heatmap showing the zygotic expressed genes (n = 601, up-regulated genes in the zygotes versus egg) commonly detected in ZM and MZ zygotes.

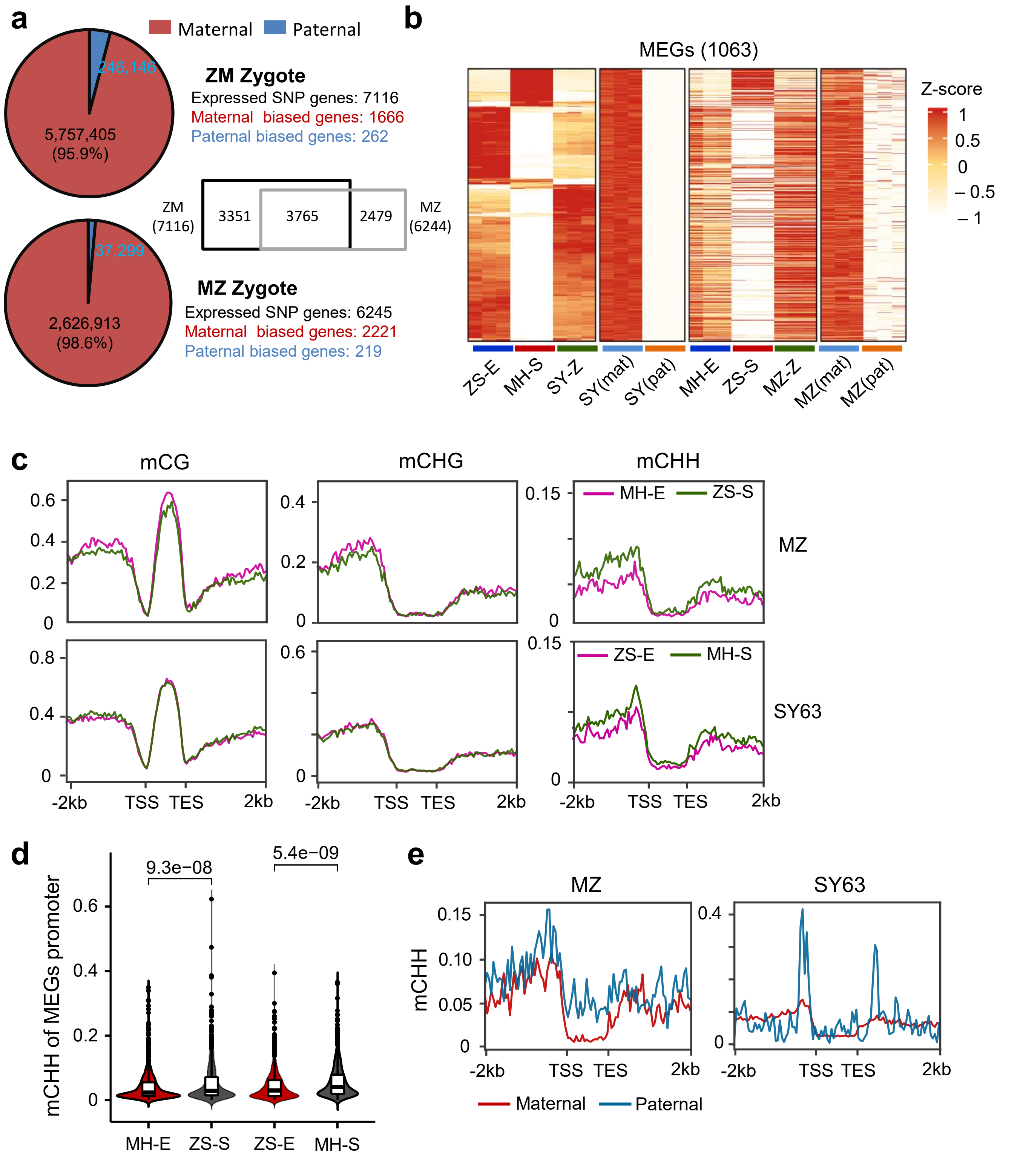

Supplementary Fig. 14. DNA methylation levels of the maternal expressed genes (MEG) in the reciprocal hybrid zygotes. (a) Percentages of the paternal and maternal allelic-specific reads from the reciprocal hybrid zygotes (upper, ZM, lower, MZ) and the numbers of expressed SNP genes. The SNP reads are mean values from the three replicates . Numbers of total, maternal biased, and paternal biased genes are indicated. Among the SNP genes in the reciprocal hybrid zygotes, 3765 genes are overlapped. **(b)** Heatmap showing expression patterns of the MEGs identified in reciprocal hybrid zygotes. **(c)** Metaplots showing DNA methylation levels of the zygotic MEGs (n = 1063) in parental gametes (MH63 egg [MH-E], ZS97 sperm [ZS-S], ZS97 egg [ZS-E], MH63 sperm [MH-S]). **(d)** Violin plots showing the CHH methylation of the zygotic MEGs (n = 1063) at promoter regions in parental gametes. *P*-value is calculated by the two-tail t-test. **(e)** Metaplots showing the parental allelic-specific methylation of the zygotic MEGs at CHH context in ZM and MZ zygotes. Source data are provided as a Source Data file.

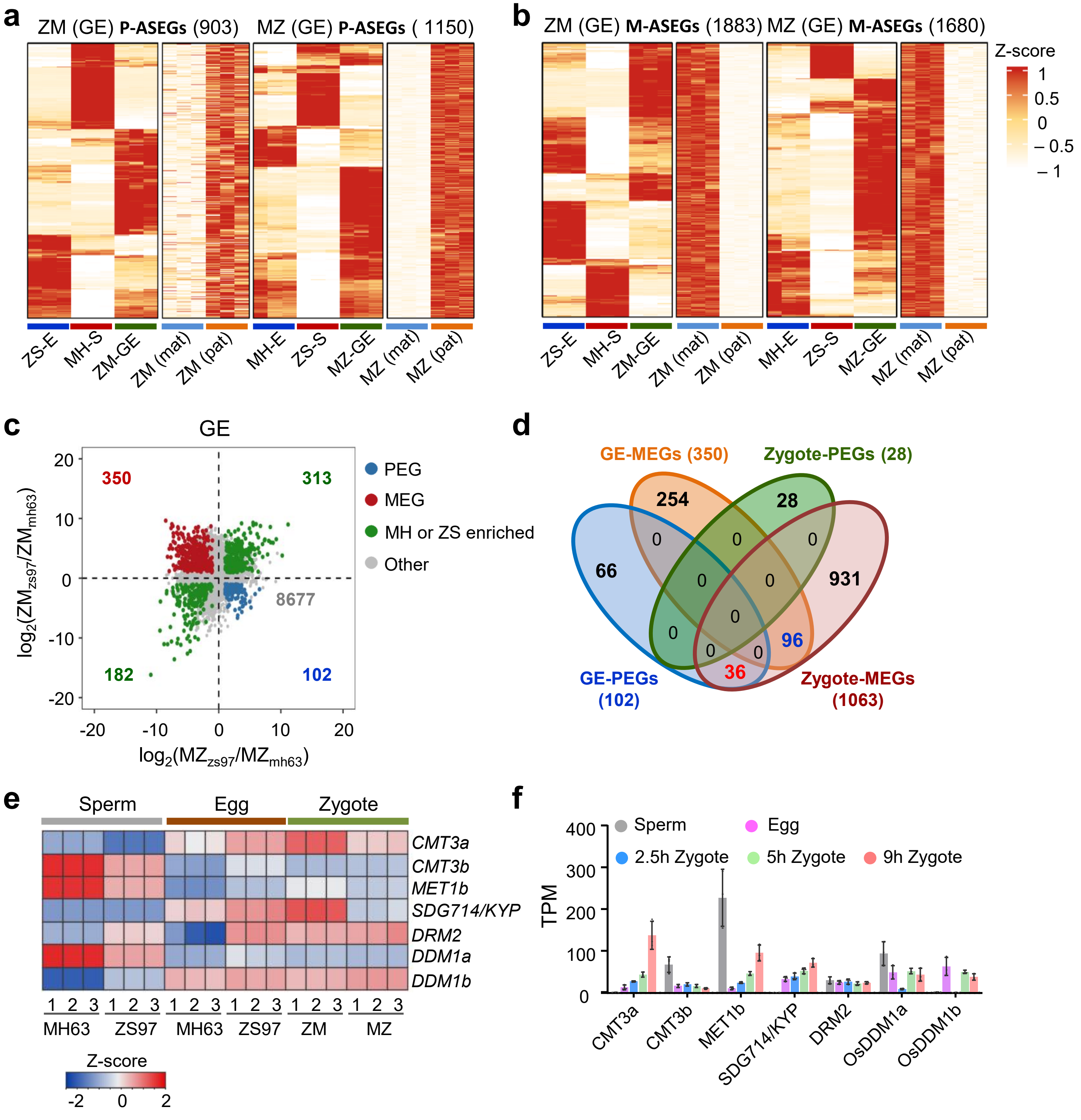

Supplementary Fig. 15. Identification of PEGs and MEGs in the reciprocal hybrid globular embryos. **(a)** Heatmap showing the paternal allelic-specific expressed genes (P-ASEGs; ZM [n = 903], MZ [n = 1150]) and **(b)** maternal allelic-specific expressed genes (M-ASEGs; ZM [n = 1883], MZ [n = 1680]) in hybrid globular embryos (GEs). **(c)** Identification of GE PEGs and MEGs by scattering analysis of the paternal ASE genes in the reciprocal hybrids (ZM and MZ). **(d)** Venn diagrams showing the overlapped PEGs and MEGs between zygotes and GEs in ZM and MZ. **(e)** Heatmap showing the expression patterns of the DNA methyltransferase genes (CMT3a/b, MET1b, KYP, DRM2, DDM1a/b) in gametes and the reciprocal hybrid zygotes of MH63 and ZS97. Three replicates are shown. **(f)** Gene expression levels of DNA methylation –related enzymes (CMT3a/b, MET1b, KYP, DRM2, DDM1a/b) in rice sperm, egg, and zygote (2.5 hap, 5 hap, 9 hap) RNA-seq data reported by Anderson et al., (2017). Error bars indicate means ± SD (n = 3). Source data are provided as a Source Data file.

Supplementary Table 1. Summary of BS-seq data.

Sample	Cell type	Replicate	Raw reads	Mapping reads	Unique mapping reads	Mapping rate	Paired reads	Duplication reads	Duplication rate	Bisulfite conversion rate	Sequencing depth (×)
MH63	Egg	1	48,712,299	37,047,721	14,412,769	38.90%	13,114,907	963,759	9.00%	98.80%	28.08
MH63	Egg	2	43,102,219	32,750,038	12,738,380	38.90%	11,614,010	1,124,370	8.83%	98.91%	24.83
MH63	Sperm	1	118,293,182	88,336,272	32,383,329	36.70%	25,660,385	6,722,942	20.76%	98.66%	66.96
MH63	Sperm	2	133,819,783	99,456,838	36,185,124	36.40%	27,851,478	8,333,645	23.03%	98.98%	75.39
MH63	Zygote	1	48,389,482	36,088,153	13,400,253	37.10%	12,032,202	1,368,051	10.21%	98.77%	27.36
MH63	Zygote	2	69,491,742	53,938,810	19,417,065	36.00%	17,071,393	2,345,671	12.08%	99.38%	40.89
ZS97	Egg	1	99,194,514	74,983,500	29,575,353	39.40%	26,638,452	2,936,898	9.93%	99.03%	56.84
ZS97	Egg	2	108,926,875	83,513,641	33,020,654	39.50%	29,528,186	3,492,467	10.58%	98.94%	63.31
ZS97	Sperm	1	59,674,897	32,536,875	10,203,668	31.40%	9,232,528	971,140	9.52%	98.94%	24.66
ZS97	Sperm	2	71,160,822	38,330,552	12,361,025	32.20%	11,009,159	1,351,866	10.94%	98.87%	29.06
ZS97	Zygote	1	58,445,027	44,771,164	18,135,148	40.50%	16,768,175	1,366,973	7.54%	98.71%	33.94
ZS97	Zygote	2	58,976,701	47,426,906	18,587,803	39.20%	17,169,368	1,418,434	7.63%	98.64%	35.95
ZH11	Egg	1	74,141,968	69,320,934	25,276,014	36.50%	16,368,122	8,907,887	35.24%	98.60%	52.55
ZH11	Egg	2	85,367,823	79,830,279	29,019,598	36.40%	17,635,050	11,384,541	39.23%	98.50%	60.51
ZM (ZS97×MH63)	Zygote	1	76,047,006	66,217,828	32,131,208	48.50%	20,409,981	11,721,223	36.48%	98.80%	50.19
ZM (ZS97×MH63)	Zygote	2	68,582,841	59,716,827	29,941,439	50.10%	19,194,312	10,747,122	35.89%	98.62%	45.27
MZ (MH63×ZS97)	Zygote	1	53,290,624	33,383,644	16,049,022	48.10%	11,763,335	4,285,686	26.70%	98.40%	25.31
MZ (MH63×ZS97)	Zygote	2	60,391,330	37,134,161	17,548,262	47.30%	12,668,605	4,879,649	27.81%	98.72%	28.15
ZMH (ZH11×MH63)	Zygote	1	59,303,272	53,923,065	21,741,671	40.30%	10,814,226	10,927,445	50.26%	98.40%	40.88
ZMH (ZH11×MH63)	Zygote	2	86,455,240	78,267,805	31,330,217	40.00%	14,414,548	16,915,668	53.99%	98.50%	59.33
ZM (ZS97×MH63)	Globular embryo	1	47,981,035	39,669,194	18,775,882	47.30%	13,830,486	4,945,391	26.34%	98.85%	30.07
ZM (ZS97×MH63)	Globular embryo	2	107,856,400	97,794,767	43,392,524	44.40%	29,456,061	13,936,456	32.12%	98.82%	74.13
MZ (MH63×ZS97)	Globular embryo	1	58,227,301	48,124,834	25,899,346	53.80%	18,646,860	7,252,477	28.00%	98.84%	36.48
MZ (MH63×ZS97)	Globular embryo	2	56,717,144	46,773,606	25,159,799	53.80%	17,971,641	7,188,150	28.57%	98.89%	35.46

Supplementary Table 2. Summary of RNA-seq data of rice gametes, hybrid zygotes and globular embryos.

Sample	Cell type	Replicate	Raw reads	Clean reads	Unique mapping reads	Mapping rate	Unique mapping rate	Sequencing depth (×)
MH63	Sperm	1	68,702,939	60,668,897	43,464,468	83.30%	71.64%	45.99
MH63	Sperm	2	53,610,062	47,475,731	33,502,893	82.92%	70.57%	35.99
MH63	Sperm	3	60,407,350	53,614,451	37,936,300	82.88%	70.76%	40.64
MH63	Egg	1	52,532,426	38,058,279	18,139,195	86.02%	47.66%	28.85
MH63	Egg	2	50,030,428	35,351,822	16,937,058	85.10%	47.91%	26.80
MH63	Egg	3	49,717,522	34,572,789	16,527,105	85.26%	47.80%	26.21
ZS97	Sperm	1	37,660,237	34,268,815	23,964,172	81.82%	69.93%	25.98
ZS97	Sperm	2	42,663,175	37,582,102	25,395,812	80.69%	67.57%	28.49
ZS97	Sperm	3	40,696,031	35,733,263	24,194,823	80.80%	67.71%	27.09
ZS97	Egg	1	25,974,715	20,437,728	18,176,156	95.12%	88.93%	15.49
ZS97	Egg	2	25,974,715	23,231,554	20,173,252	93.57%	86.84%	17.61
ZS97	Egg	3	24,870,195	18,321,762	16,205,559	94.64%	88.45%	13.89
ZM (ZS97×MH63)	Zygote	1	48,049,718	38,097,665	28,078,508	90.83%	73.70%	28.88
ZM (ZS97×MH63)	Zygote	2	40,086,078	29,621,174	21,938,319	90.79%	74.06%	22.45
ZM (ZS97×MH63)	Zygote	3	43,045,792	33,745,559	24,906,428	90.45%	73.81%	25.58
ZM (ZS97×MH63)	GE	1	40,232,511	28,753,447	11,165,964	67.37%	38.83%	21.80
ZM (ZS97×MH63)	GE	2	41,158,039	28,523,186	11,288,799	66.80%	39.58%	21.62
ZM (ZS97×MH63)	GE	3	32,752,040	23,063,316	9,704,605	70.36%	42.08%	17.48
MZ (MH63×ZS97)	Zygote	1	43,098,456	30,432,243	14,538,079	73.03%	47.77%	23.07
MZ (MH63×ZS97)	Zygote	2	55,708,018	37,123,363	17,588,619	70.68%	47.38%	28.14
MZ (MH63×ZS97)	Zygote	3	48,383,975	32,007,194	14,578,072	68.98%	45.55%	24.26
MZ (MH63×ZS97)	GE	1	58,280,095	33,561,837	16,116,131	75.72%	48.02%	25.44
MZ (MH63×ZS97)	GE	2	51,975,612	30,255,395	15,141,778	76.55%	50.05%	22.93
MZ (MH63×ZS97)	GE	3	49,055,554	32,587,059	16,504,772	79.01%	50.65%	24.70
