# Supplementary material for: Paternal DNA methylation is remodeled to maternal levels in rice zygote
Source: Nat Commun. 2023 Oct 18;14:6571. doi: 10.1038/s41467-023-42394-0 (PMC10584822; doi:10.1038/s41467-023-42394-0)
Supplement: Supplementary file 4 — Description of Additional Supplementary Files [file 41467_2023_42394_MOESM4_ESM.pdf]

Description of Additional Supplementary Files

Supplementary Data 1. The differentially methylated genes (DMGs) commonly found in inbred and hybrid zygotes relative to gametes.

Supplementary Data 2. The up-regulated and downregulated genes commonly detected in the reciprocal hybrid zygotes.
